# Radical Acceptance of Reality: Putting DBT®-A Skill Groups Online During the COVID-19 Pandemic: A Qualitative Study

**DOI:** 10.3389/fpsyt.2022.617941

**Published:** 2022-04-25

**Authors:** Mercedes M. Bock, Theres Graf, Verena Woeber, Oswald D. Kothgassner, Arne Buerger, Paul L. Plener

**Affiliations:** ^1^Department of Child and Adolescent Psychiatry, Medical University of Vienna, Vienna, Austria; ^2^Psychosocial Services, Vienna, Austria; ^3^Department of Clinical Psychology and Psychotherapy, Wiener Gesundheitsverbund, University Clinic AKH, Vienna, Austria; ^4^Department of Child and Adolescent Psychiatry, Wiener Gesundheitsverbund, University Clinic AKH, Vienna, Austria; ^5^Department of Child and Adolescent Psychiatry, Psychosomatics and Psychotherapy, University Hospital of Wuerzburg, Würzburg, Germany; ^6^Department of Child- and Adolescent Psychiatry and Psychotherapy, Medical University of Ulm, Ulm, Germany

**Keywords:** DBT-A, skills group, COVID-19, emotion dysregulation, teletherapy, online, video

## Abstract

**Introduction:**

Emotion dysregulation is a common challenge pertaining to numerous psychiatric disorders in adolescence and is associated with increased morbidity and mortality. Dialectical Behavior Therapy for Adolescents (DBT®-A) has been shown to be an effective treatment, especially in the reduction of self-harm and suicidality. Measures in relation to the COVID-19 pandemic set strict limits on physical contacts with patients. In order to continuously provide evidence-based specialized care to patients suffering from emotion dysregulation, we offered two online DBT®-A skill groups in a video-group-call format.

**Objective:**

We aimed at assessing our online DBT®-A skills groups, collect according up- and downsides, and form a basis for advancement of this form of treatment provision. Also, the impact of the COVID-19 pandemic on patients was assessed.

**Methods:**

A physical DBT®-A skill group was switched to a video-group-call format and a second group was initiated *de novo online*. After five sessions, patients engaged in structured group discussions to reflect experiences. Discussion content was analyzed via Inductive Category Formation within the Framework of Qualitative Content Analysis.

**Results:**

Patients unanimously found the COVID-19 pandemic challenging, but also reported differentially on its impact. Downsides were balanced by subjective “gains” in time and a perceived reduction in stress. Technical problems of the online format were discussed, but did not limit the positive experience of still receiving treatment. Patients of both online DBT®-A skill groups valued the offer, felt connected, and reported benefits from the treatment. The *transition* group additionally discussed changes in structure and content of the group sessions after the switch to online meetings and reflected differential functions of the group.

**Discussion:**

Although the sample size is small, and conclusions are drawn from Inductive Qualitative Content Analysis, the presented results are of interest. In our investigation, video-group-calls were both safe and beneficial for patients. This alternative to physical meetings is not only interesting for further waves of the current pandemic but also for service provision in remote areas with limited access to specialized care. Further research is needed to challenge and refine our results and to explore extensions to “basic” video-group-calls, such as “break-out sessions,” blended therapy, or real-time supervision within an online session.

## Introduction

Emotion dysregulation is seen as a trans-diagnostic factor with negative impact on various domains of psychopathology in adolescents (1), although specifics could be identified in the subgroup of patients suffering from borderline personality disorder (BPD) (2). Deficits in emotion regulation are associated with social impairment (3) and, consequently, contribute negatively to the level of psychosocial functioning. Additionally, typical behavioral manifestations of emotion dysregulation, such as self-harm, suicidal ideation, eating disorder behaviors, or substance (ab)use, are associated with increased morbidity and mortality (4–7). According to international standards, Dialectical Behavior Therapy for Adolescents (DBT®-A) is an evidence-based treatment for these patients, especially concerning the reduction of self-harm (8–11). DBT-A is a manualized treatment tailored for application in outpatient settings (12–14). Full DBT®-A treatment comprises weekly individual sessions, concurrent participation in a DBT®-A skills group, telephone coaching, consultation team, and family work (13, 15). Specific strategies taught within the skills group component of DBT®-A are mindfulness, distress tolerance, interpersonal effectiveness, emotion regulation, and “walking the middle path” (strategies connecting teens and caregivers).

In integrating the above with results from first observations on the impact of the COVID-19 pandemic on patients suffering from mental health disorders, the continued provision of evidence-based care is of utmost importance. So far, data clearly indicate disproportional consequences of the COVID-19 pandemic (16–18), even a potentially increased suicide risk (18), and an increase of adversities (19) in patients suffering from mental health disorders.

Owing to restrictions set to ensure minimum spreading of SARS-CoV-2, not only in everyday life, but also in clinical routine suffered critical cuts. Limits especially concerned physical contacts, which were reduced to an absolute minimum (i.e., emergency presentations in the psychiatric context). Given the need to continuously serve patients despite these restrictions, a lot of effort went into provision of services through other means of care, such as telehealth interventions (20). In the need of the moment and given changes in several legal regulations, many departments of child and adolescent psychiatry conducted an *ad hoc* switch from face-to-face to teletherapy to continuously serve their current outpatients (21). Although the preexisting body of literature on telemental health, many countries stepped into this venture without preparation because of lack of legal frameworks and lacking prior approval on the conductance of teletherapy by legal authorities and professional organizations (22). Even in countries with regulations and formal licensing procedures, such as the United States, many therapists were unexperienced in using telemental health (23). Sampaio et al. could show that around half of the mental health professionals in their survey were concerned about data safety when using telemental health.

In order to ensure continuation of high-quality, evidence-based services, especially in expectance of further waves of the current pandemic, we aimed at systematically evaluating patients' experiences with our DBT-A skill group in the newly established video group call format via structured group discussions and consecutive qualitative analyses^1^. Sampaio et al. argued that around 50% of mental health professionals expressed concerns about their ability to handle emergency situations in a telemental health setting (23), which is of special interest for our research because the population investigated herein were “at risk” patients (self-harm, suicidality).

Little et al. (24) argued that qualitative methodology is suitable to address and assess patients' perceptions of DBT®. Since presentation, discussion and training of behavioral, cognitive, and emotional skills serve as one of the main therapeutic tools in overcoming pervasive emotion dysregulation (25, 26), we decided to focus on this component. Also, Linehan et al. (27) could show that skills training alone leads to better results concerning the frequency of self-harm than individual DBT®, as well as overall similar rates concerning other symptoms of BPD addressed within therapy (e.g., suicidal ideations, suicide attempts). The aim of this small open trial was, thus, to assess practicability and record potential adverse effects of DBT®-A skills training in a video group call format. Thoughts and ideas expressed by our patients, as well as insights gained in the process, were deemed to form the base for further steps, especially concerning positive lessons, but also flipsides of this form of treatment conductance. Although this is especially relevant in case of further waves of the COVID-19 pandemic, results might also be helpful concerning potential applications in other situations, such as service provision in remote areas with limited access to specialized care.

## Materials and Methods

### Participants

Of five patients participating in our *physical* group, four decided to continue online. All four patients completed the online “cycle.” Although all patients gave consent to study participation, only three engaged in the investigative group discussion (one patient had a colliding appointment).

Six patients showed interest and started in our second *de novo online* group. Of these six patients, one dropped out after two sessions because she felt the content was not the right fit for her, and one left the group after three sessions because of repeated technical problems (unstable internet connection). The remaining four patients gave consent to study participation, but only three were online at the time of the investigative group discussion. One patient had technical problems during the discussion and, thus, could not continuously contribute. Concerning dropouts, we saw no difference to our *physical* groups. The dropout rates we observed in our sample were at the lower end of rates reported in other studies (28, 29). Also, dropout rates were equal in both online groups, which is of interest because the module, and thus, content differed. This is in line with the observations of Landes et al. (28).

All patients received concurrent psychiatric treatment (case management and psychopharmaceuticals) and individual psychotherapy before, as well as during the COVID-19 pandemic (during lockdown per telephone or video call). Patients were between 14 and 18 years old and mainly female. The leading diagnoses were BPD, depressive disorder, or anxiety disorder (diagnoses according to ICD-10). Non-BPD patients met at least three BPD criteria.

### Procedure

Given the need to continuously offer specialized care in times of COVID-19-associated restrictions on physical contact, our clinic's DBT®-A team decided to create an online offer. DBT®-A skill group sessions were conducted in a video group call format. Skype for Business (Skype Communications SARL, Luxembourg) was used as platform conforming to Austrian data safeguarding measures. The respective session link was always sent via email a day before the session.

Patients of the current DBT®-A skill group and patients new to DBT®-A, as well as all respective parents/guardians, were asked individually, per telephone or video call, whether they would participate in an online format. Two separate DBT®-A online skill groups were offered. Group 1 was transited from an existing *physical* group to online meetings after a COVID-19-associated break of 3 weeks (time needed for organization, preparation of technology, checking compliance to data safety regulations). Group 2 was initiated *de novo online* 3 weeks after the onset of Group 1. Lockdown measures were still active at the time.

Two group discussions, conceptualized to evaluate each of the online DBT®-A skill groups, were conducted online via Cisco WebEx (Webex Communications Deutschland GmbH, Germany).

Ethical approval for this investigation was granted by the institutional review board of the Medical University of Vienna (#1540/2020), and patients and guardians gave written consent prior to study participation.

### Description of Treatment

DBT®-A skill group contracts were adapted so as to suit online sessions and were signed by all patients. The following methods and materials were used: (1) presentation of content by skill trainers, (2) group discussions engaging all patients, (3) chat function where applicable (e.g., group tasks), (4) worksheets (distributed via email), (5) shared files for continued group work (e.g., collective PowerPoint presentations for collection of skills manageable within the set restrictions, which were conducted with enthusiasm and creativity by the patients and the trainers, and which were presented and discussed in the next session), and (6) peer feedback. Session structure was defined so as to follow the same basic routine for both groups, although specific content varied between the two groups. This variation was owed to the fact that the first of the two online DBT®-A skill groups was transited from an existing *physical* group and, thus, already had prior knowledge on a range of skills, while the second group started *de novo online*. In order to follow the need of the moment, we decided to align content around the current module (group 1 → emotion regulation, group 2 → distress tolerance), but integrate specific content according to patients' current needs, thus, sometimes giving the group the lead and reacting spontaneously including skills from other modules. Basic session structure and specific content per group and appointment for the first five sessions (evaluation after session 5) are listed in Tables 1, 2, respectively. Upon onset of the online format, the DBT®-A team was unsure about session length (presence groups lasted for 90 min including a 15-min break), so that this was openly discussed with participants from group 1, who by then all had experience with distance learning. Consensus was reached at a session length of 60 min. In order to ensure patient safety, each patient had to name a contact (mostly parent) beforehand, who could be reached in case of emergency (this emergency procedure actually never had to be used). Contact details of patients and emergency contacts were kept “ready to use” by both skill trainers. Additionally, patients were informed that in case of emergency (acute tension, need for immediate support/action) one of the two trainers would continue the session alone, and the other would be available in a separate online meeting or via telephone.^2^ Sessions were prepared in a separate, weekly online meeting by the two DBT®-A skill trainers. Each session was followed by a reflection of the trainers directly after the end of the respective session. Supervision via telephone conference by an external supervisor was well-established and continued during the pandemic crisis and the according DBT®-A online format.

**Table 1:** Session structure.

**Topic**	**Time**
Greetings and quick evaluation of acute needs	5'
Mindfulness	5'
▪ Exercise ▪ Reflection	(2') (3')
Collection of “hot topics” (specific needs in relation to the dynamic of the COVID-19 pandemic)	5'
Recap	10'
▪ “Challenge” (therapeutic homework) ▪ “Skills of the week” (top skills applied)	(5') (5')
New content (following the current module, integrating specific needs addressed during collection of “hot topics”)	20'
Assignment and explanation of “challenge” for the upcoming	5'
Mindfulness in the sense of reflection of the current session	5'
Buffer	5'

**Table 2:** Specific session content.

**Appointment**	**Content**
**Group 1 (*****transition*** **group)**	
1	▪ “Update” after pandemic-associated 3-week break ▪ Collection of current needs ▪ Explanation of structure of online sessions ▪ Short discussion about session length ▪ Evaluation of current survival skills (idea that some might not be applicable during lockdown) ▪ Group discussion (recap from prior modules) concerning survival skills “challenge” → revision of individual crisis survival kits
2	▪ Introduction to module “emotion regulation” ▪ Understanding emotions ▪ Group discussion concerning specific emotions ▪ Specific skill: *ABC PLEASE* ▪ Specific skill: *accepting reality* “Challenge” → practice *ABC PLEASE*
3	▪ Specific skill: *opposite action* ▪ Specific skill: *focusing on positive events* ▪ Group work (using the chat)—collection of current (or potential) positive events ▪ Mindfulness story (“bean exercise”) “Challenge” → collection of “beans” and contribution to shared file (collection of positive events)
4	▪ Specific skill: *check the facts* ▪ “Guide to” dialectics ▪ Group discussion concerning specific skill: *thinking and acting dialectically* “Challenge” → *re-phrasing dialectically*
5	▪ Specific skill: *wave skill* ▪ “Expert” discussion (one patient already had experience with the wave skill) ▪ “Guide to” validation ▪ Specific skill: *validation of self* (followed by group discussion) “Challenge” → “fairness” and *validation of self*
**Group 2 (*****de novo online*** **group)**	
1	▪ Introduction/getting to know each other online ▪ Explanation of structure of online sessions ▪ Collection of current needs ▪ Collection of strategies applied so far (and labeling of specific skills) ▪ “Guide to” understanding tension and early warning signs ▪ Introduction to survival skills ▪ Specific skill: *self-sooth with the six senses* ▪ specific skill: *PLEASE* “Challenge” → experimenting with at least one (max 3) six sense skills at medium tension, observation of *PLEASE*
2	▪ Recap of early warning signs ▪ “Hot chair” ▪ “Guide to” mindfulness ▪ What and how skills ▪ Exercise: description of picture ▪ Specific skill: *IMPROVE the moment* “Challenge” → experimenting with at least one further six *sense skill, IMPROVE the moment*, collection of individual early warning signs
3	▪ Coordinative exercise ▪ Specific skill: *accepting reality* ▪ Specific skill: *focusing on positive events* ▪ Group work (using the chat)—collection of current (or potential) positive events ▪ Mindfulness story (“bean exercise”) “Challenge” → collection of “beans,” contribution to shared file
	(collection of positive events), “first draft” of individual crisis survival kits
4	▪ Coordinative exercise ▪ Crisis survival kits ▪ Specific skill: *wise mind ACCEPTS* ▪ Recap of specific skill: *PLEASE* Challenge” → *PLEASE* and *wise mind ACCEPTS*
5	▪ “Cognitive” exercise ▪ Discussion of vulnerability ▪ “Guide to” validation ▪ Specific skill: *validation of self* (followed by group discussion) “Challenge” → “fairness” and *validation of self*

### Study Design

To our knowledge, a video group call format of a DBT® (-A) skill group has not yet been evaluated, so this investigation was set up as an exploratory pilot study. Due to the novelty of the situation, only limited prior knowledge on its effects on adolescents suffering from mental health disorders (especially emotion dysregulation, self-harm, and suicidal ideations), and a limited number of participants, we opted for a purely qualitative evaluation in the form of online group discussions and according analyses. Separate discussions were planned for each of the two online DBT®-A skills groups at our clinic after five sessions of skill training. Details of the study were explained to patients and guardians separate from details concerning the skill training sessions, and written informed consent was obtained. The group discussion sessions were configurated by a qualified research fellow and senior resident in Child and Adolescent Psychiatry, who was not involved in the treatment of the respective patients. Session links were sent per email to all participants by one of the skill trainers. At the beginning of the group discussion sessions, both skill trainers were present and introduced the moderator of the discussion. The skills trainers then logged out of the respective session and stayed available as background support per telephone or video call for any acute situations or technical problems. Immediately after the end of the respective discussions, the moderator left the meeting, and the skill trainers took over to resume and round up.

### Measurements

Discussions were conceptualized, moderated, and analyzed by a qualified research fellow and senior resident in Child and Adolescent Psychiatry, who was not involved in the treatment of the respective patients. The format of a group discussion, due to the shared background of the patients following the ideas of Bohnsack (30), was chosen because perceptions and opinions of participants were interpreted as being results of the process of the groups *per se* and were, thus, deemed to be best accessible via discussion within the established groups. Discussions followed the structure *stimulus*→*discussion* → “*exmanent” questioning*^3^→*directive phase*→*protocol*. Stimuli are understood as input material to start off the process of discussion. Most frequently, statements, videos, audio files, or pictures are being used. The main discussion is understood as exchange between the participants without intervention of the moderator. When the discussion is “exhausted,” i.e., there are no more novel contributions or long pauses, the moderator steps in to ask questions that have not been addressed by the participants, but are of relevance for the research in question. The directive phase of the discussion serves clarification; contradictory statements are being picked up by the moderator, and participants are asked for further input.

The stimulus presented in this study were verbal captions of the current situation and varied slightly between the two groups, so as to additionally encompass the fact that the *transition* group already had a good working alliance before the start of the online treatment, while the *de novo online* group had to manage this process online.

### Data Analysis

In light of the novelty of the situation and the consequent lack of prior information, in-depth qualitative analysis of discussion content was open and exploratory, applying Inductive Category Formation within the framework of Qualitative Content Analysis (Mayring) (31). Analysis was supported by the standard computerized qualitative data analysis tool ATLAS.ti 8 (ATLAS.ti Scientific Software Development GmbH, Berlin, Germany). A summary description of the according analytical steps is presented in Table 3.

**Table 3:** Summary description of Inductive Category Formation (Qualitative Content Analysis) (31).

**Step**	**Content**
Step 1	Research of literature (theoretical background), formulation of research question(s)
Step 2	Establishment of selection criterion, definition of categories, and level of abstraction
Step 3	Working through the material “line by line” → either formulation of new categories or subsumption
Step 4	Revision of categories and rules after 10%−50% of the material
Step 5	Final working through the material
Step 6	Building of main categories
Step 7	Going back to the material to back-check categories
Step 8	Final results and interpretation

## Results

Content of the group discussions varied between the two groups, whereby group 1 engaged in a more in-depth discussion on specifics of teletherapy and benefits from the skill group, in general, and especially in times of the COVID-19 pandemic, while group 2 stayed on a more superficial level and rather focused on differential impacts of the pandemic.

### Perceived Impact of the Pandemic

Patients of both groups unanimously agreed upon the challenging character of the current situation and the need to engage in adjustment processes [“It, like, happened all at once, it was simply there, it was so overwhelming.”].^4^ Both groups expressed up- and downsides of lockdown measures and associated restrictions. Main themes on the positive side were the feeling of having more time (for relaxation, for reflection, for the family) [“All of a sudden I had time, time that I never had before.”] and experiencing less stress. Patients also mentioned the perception of now valuing things more (“little” things, freedom, contacts) [“I've learned to appreciate every-day life things a lot more.”], and the feeling of confronting and experimenting more (affective states, emotions and respective skills); in their view mainly attributed to fewer distractions [“The situation implied that I could not run away from my feelings that much, because I just had less distraction. Obviously, that wasn't always comfortable, but it made me get to know my emotions and deal with them.”]. Flipsides named were restrictions concerning typically applied coping strategies, boredom, self-isolation [“It was just so easy to self-isolate.”], decreased mood, less drive, and the missing of physical contacts (both in the sense of seeing them for real and being touched) with friends.

### Technical Issues

Complaints about technical problems and specific challenges of teletherapy as such, especially video calls, did not relate to group membership. Only one patient (group 1) expressed frustration concerning problems with the internet connection, but said that this was easily circumventable by intermittent use of the chat function. Additionally, one patient from group 1 and one patient from group 2 expressed overall discomfort. Issues raised were irritation due to constantly seeing oneself (video in small display window) and the feeling of potentially being overheard by family members and consequently being less open. At the same time, both of these patients stated that they valued the offer as an alternative to not being able to receive specialized treatment at all. Both clearly stated to have benefitted a lot and would just prefer real-life contacts. On the other hand, one patient mentioned feeling more comfortable with the video call setting, due to not having to hide wounds and scars, because only her head and chest could be seen. This patient added that she found it easier to apply skills in case of distress during the sessions because of the opportunity to turn off the camera for a short time.

### Perceived Benefits

Patients reported on having learned to recognize and label different emotions and affective states, as well as behavioral patterns. They also valued the discussion of specific strategies to deal with according distress. Patients from both groups mentioned that they were able to amplify their coping repertoire. Additionally, the interactive character and the chance to give and receive peer feedback, which patients perceived as strongly supporting transfer into everyday application of specific skills, was mentioned as beneficial [“It was so positive that we could discuss ideas and provide potential solutions within the group, so we really could give and take.”].

**Figure 1:**
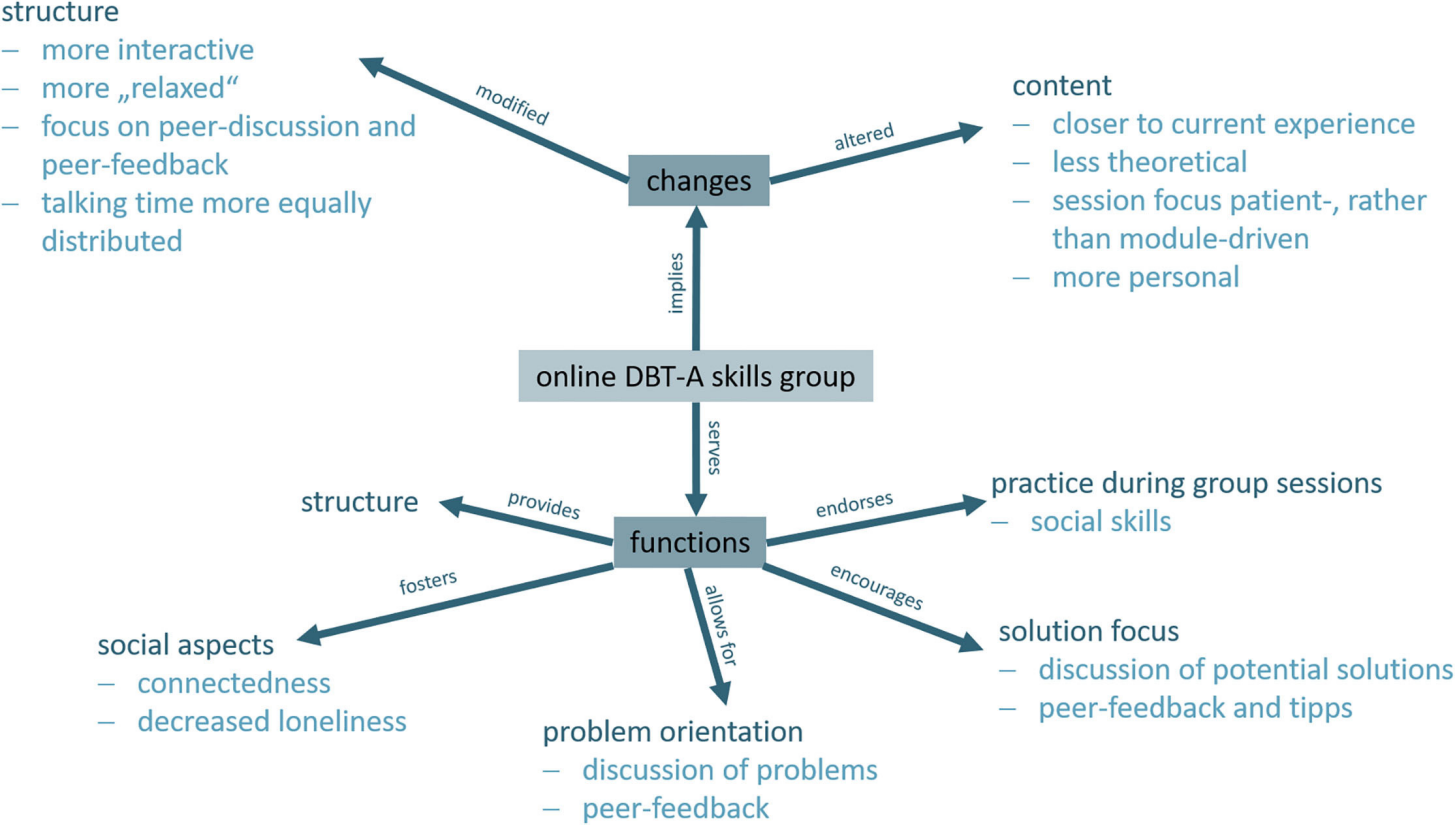
Related themes elaborated during Inductive Category Formation.

Reports from group 1 were more elaborated and integrated perceptions of differential changes in the structure and content of group sessions after the transition to the online format, as well as individually perceived functions of the group as such. To use the words of one of the patients, the group provided “familiarity within the unfamiliar.” Details of the compiled categories are depicted in Figure 1. To summarize the analysis, patients of the *transition* group benefitted from continuation of established therapy and assigned a range of subjective functions of the group as such. The transition into an online setting worked for all of these patients and was reflected in the context of “change”; situational adaptions were mirrored in perceptions of changed structure and content of the group sessions. One of our patients summarized: “The mundane structure I was used to and, due to Corona, did not have anymore, was filled with this subtle but very helpful appointment.”

Concerning the level of psychosocial functioning, all patients managed to stay socially integrated (with all limits imposed by the set restrictions) and to keep up with school, i.e., all patients made their grades; one even the school ending exam (“Matura”).

## Discussion

The COVID-19-pandemic had, and still has, a huge impact on people worldwide and not only demanded adaptions from individuals and systems but also required immense situational adjustment. Specific subpopulations, such as individuals suffering from psychiatric disorders, have been suspected to have an increased risk of deterioration in their mental health status, although limited data are available differentially evaluating potential consequences of the crisis (32, 33). All else put aside, current outpatients were confronted with the fact that measures set to ensure minimum spreading of SARS-CoV-2 imposed considerable changes in the availability of established treatment, ranging from treatment pause to an *ad hoc* switch to teletherapy (e.g., telephone, video calls). Data on the provision of DBT® and consequences for patients suffering from BPD during COVID-19 suggest serious consequences, especially reduced numbers treated and changes in structure and content of therapy (34). Although teletherapy generally seems to be beneficial (35, 36), many countries were unprepared for it due to lacking legal frameworks, lacking prior approval on the conductance of teletherapy by legal authorities and professional organizations, and lacking clarity concerning cost coverage or reimbursement (22). Interestingly, even in countries with preexisting regulations on tele (mental) health, such as the United States, many mental health professionals had not used telemental health before the outbreak of the COVID-19 pandemic, whereby data safety and a lack of preparedness for handling emergency situations seem to have been the main concerns (23). Regulations in our country precluded video-calls as a legitimate treatment alternative so that the patients assessed in this study had no prior experience with teletherapeutic offers. When offered the option to participate in an internet-based video call format, only one patient from our ongoing *physical* group declined, so that we could assess the transition aspect—face to face switched to teletherapy—in this established group. Additionally, we invited patients new to us and to DBT-A to join a second *de novo online* skill group, which enabled us to assess the process of forming a group online and explore challenges in conveying DBT®-A skills via this format.

Concerning participation, we lost two patients due to dropouts. This number did not differ from recorded dropouts in our *physical* groups and is in line with rates reported in other studies (29).

Although this study is exploratory, and results are drawn from a small sample, we provided evidence for a differentiated view on the impact of the COVID-19 pandemic on adolescent psychiatric outpatients. Although perceiving the situation as challenging, patients not only named downsides but also numerous positive consequences during lockdown. Concerning technical aspects, the situation was challenging for both therapists and patients, although only one patient elaborated this aspect in the group discussion. As skill trainers, we decided to include the chat function of our video call platform to keep collaboration and exchange going in case of problems, which was valued by participants (e.g., a note that a patient will turn off the camera in case of the need to apply skills during the session, unstable internet connection, or quality problems with video and sound). Patients of the *transition* group could also be evaluated concerning the perception and acceptance of changes in content and structure. The feedback was very positive concerning both changes in content and structure. Patients stated that the sessions became more interactive than the physical meetings, and they especially valued extensive peer discussions and peer feedback, which we encouraged in order to give room for group exchange on pandemic-related topics. This “togetherness” was also directly addressed in the group discussions. Interestingly, also group 2, who only got to know each other online, stated feeling “connected” and also put emphasis on the group providing an anchor, something to hold onto.

Regarding patient safety, a meeting was arranged, and in consent with the patient, the patient's individual therapist was contacted in order to ensure continued treatment. One patient dropped out after the third session because her internet connection failed repeatedly, and she was neither able to interact, nor to follow the content.

Generalization of results is limited by the sample size and characteristics, the exploratory character, and the consequently applied qualitative methodology. Also, only five sessions of the online DBT®-A skill group have been evaluated.

Despite the abovementioned limitations, in our assessment, DBT®-A skill training via internet-based video group call proved to be safe and beneficial for adolescents with emotion dysregulation. This result is especially relevant since many therapists are concerned about their ability to handle emergency situations in a telemental health setting (23). Our results indicate that an online format might be an alternative to face-to-face group sessions if these are not possible. This is obviously interesting concerning future situations of quarantine, especially further waves of the current pandemic, but potentially also for service provision in remote areas, where highly specialized treatment often is not available.

In spite of the encouraging character of these results, further research is necessary to challenge and refine our findings, especially in the sense of a direct comparison of online vs. face-to-face DBT®-A skill groups, to assess suitability as alternative to or augmentation of face-to-face treatment in a more general context aside from quarantine. Despite the availability of smartphones and internet connection, technical difficulties, especially concerning speed and stability of the connection, have been raised by our patients, so that investment in enhancing availability of adequate technologies is necessary. Additionally, extensions of the video group call format, such as “break-out sessions” (i.e., parallel video sessions) for teamwork, real-time inclusion of an external supervisor or blended therapy has to be considered, in order to further test the potential of this format. Also, combinations with other technologies, such as virtual reality (e.g., Virtual Reality Mindfulness), should be explored within *online* DBT®(-A) (23).

## Data Availability Statement

The raw data supporting the conclusions of this article will be made available by the authors, without undue reservation.

## Ethics Statement

The studies involving human participants were reviewed and approved by Institutional Review Board of the Medical University of Vienna. Written informed consent to participate in this study was provided by the participants' legal guardian/next of kin.

## Author Contributions

MB, TG, and PP conceptualized the study. MB and VW conveyed the DBT-A treatment analyzed. TG planned and conducted the focus groups and according qualitative data analyses. MB, AB, OK, and PP all contributed to writing the manuscript. All authors contributed to the article and approved the submitted version.

## Conflict of Interest

MB has received research funding from the Medical Scientific Fund of the Mayor of the City of Vienna. PP has received research funding from Lundbeck and Servier. He has received a speaker's honorarium from Shire. The remaining authors declare that the research was conducted in the absence of any commercial or financial relationships that could be construed as a potential conflict of interest.

## Publisher's Note

All claims expressed in this article are solely those of the authors and do not necessarily represent those of their affiliated organizations, or those of the publisher, the editors and the reviewers. Any product that may be evaluated in this article, or claim that may be made by its manufacturer, is not guaranteed or endorsed by the publisher.

## Footnotes

^1^All patients also had individual therapy, which was not evaluated within this study.

^2^We twice had to make use of this option. In both cases (once in group 1 and once in group 2), patients could be reached on their smartphones and rejoined the running session.

^3^“Exmanent” questioning translates into the moderator actively asking about topics that have not yet been addressed, but are relevant to the research question(s).

^4^For illustration, patient quotes are inserted in square brackets.

## References

[1] McLaughlin KA Hatzenbuehler ML Mennin DS Nolen-Hoeksema S. Emotion dysregulation and adolescent psychopathology: a prospective study. Behav Res Ther. (2011) 49:544–54. 10.1016/j.brat.2011.06.003 21718967 PMC3153591

[2] Ibraheim M Kalpakci A Sharp C. The specificity of emotion dysregulation in adolescents with borderline personality disorder: comparison with psychiatric and healthy controls. Borderline Personal Disord Emot Dysregul. (2017) 4:1. 10.1186/s40479-017-0052-x 28078089 PMC5223469

[3] Bunford N Evans SW Langberg JM. Emotion dysregulation is associated with social impairment among young adolescents with ADHD. J Attent Disord. (2018) 22:66–82. 10.1177/1087054714527793 24681899

[4] Ajetunmobi O Taylor M Stockton D Wood R. Early death in those previously hospitalised for mental healthcare in Scotland: a nationwide cohort study, 1986-2010. BMJ Open. (2013) 3:e002768. 10.1136/bmjopen-2013-002768 23901025 PMC3731727

[5] Scott LN Pilkonis PA Hipwell AE Keenan K Stepp SD. Non-suicidal self-injury and suicidal ideation as predictors of suicide attempts in adolescent girls: a multi-wave prospective study. Compr Psychiatry. (2015) 58:1–10. 10.1016/j.comppsych.2014.12.011 25595520 PMC4369422

[6] Ribeiro JD Franklin JC Fox KR Bentley KH Kleiman EM Chang BP. Self-injurious thoughts and behaviors as risk factors for future suicide ideation, attempts, and death: a meta-analysis of longitudinal studies. Psychol Med. (2016) 46:225–36. 10.1017/S0033291715001804 26370729 PMC4774896

[7] Wolff JC Thompson E Thomas SA Nesi J Bettis AH Ransford B. Emotion dysregulation and non-suicidal self-injury: a systematic review and meta-analysis. Eur Psychiatry. (2019) 59:25–36. 10.1016/j.eurpsy.2019.03.004 30986729 PMC6538442

[8] Glenn CR Esposito EC Porter AC Robinson DJ. Evidence base update of psychosocial treatments for self-injurious thoughts and behaviors in youth. J Clin Child Adolesc Psychol. (2019) 48:357–92. 10.1080/15374416.2019.1591281 31046461 PMC6534465

[9] Kothgassner OD Robinson K Goreis A Ougrin D Plener PL. Does treatment method matter? A meta-analysis of the past 20 years of research on therapeutic interventions for self-harm and suicidal ideation in adolescents. Borderline Personal Disord Emot Dysregul. (2020) 7:9. 10.1186/s40479-020-00123-9 32426138 PMC7216729

[10] McCauley E Berk MS Asarnow JR Adrian M Cohen J Korslund K. Efficacy of dialectical behavior therapy for adolescents at high risk for suicide: a randomized clinical trial. JAMA Psychiatry. (2018) 75:777–85. 10.1001/jamapsychiatry.2018.1109 29926087 PMC6584278

[11] Mehlum L Ramleth RK Tørmoen AJ Haga E Diep LM Stanley BH. Long term effectiveness of dialectical behavior therapy versus enhanced usual care for adolescents with self-harming and suicidal behavior. J Child Psychol Psychiatry. (2019) 60:1112–22. 10.1111/jcpp.13077 31127612

[12] Linehan MM. DBT® Skills Training Manual. 2nd ed. New York: Guilford Press; (2015).

[13] Rathus JH Miller AL. Dialectical behavior therapy adapted for suicidal adolescents. Suicide Life Threat Behav. (2002) 32:146–57. 10.1521/suli.32.2.146.24399 12079031

[14] Auer AKv Bohus M editors. Interaktives Skillstraining für Jugendliche mit Problemen der Gefühlsregulation (DBT-A®). Das Therapeutenmanual. 1 ed. Stuttgart, Germany: Schattauer; (2017).

[15] Miller AL Rathus JH Linehan MM. Dialectical Behavior Therapy® with Suicidal Adolescents. New York: The Guilford Press;. (2017) 346 p.

[16] Cui Y Li Y Zheng Y Chinese Chinese Society of C Adolescent P. Mental health services for children in China during the COVID-19 pandemic: results of an expert-based national survey among child and adolescent psychiatric hospitals. Eur Child Adolesc Psychiatry. (2020) 29:743–8. 10.1007/s00787-020-01548-x 32394092 PMC7213539

[17] Fegert JM Vitiello B Plener PL Clemens V. Challenges and burden of the Coronavirus 2019 (COVID-19) pandemic for child and adolescent mental health: a narrative review to highlight clinical and research needs in the acute phase and the long return to normality. Child Adolesc Psychiatry Ment Health. (2020) 14:20. 10.1186/s13034-020-00329-3 32419840 PMC7216870

[18] Hao F Tan W Jiang L Zhang L Zhao X Zou Y. Do psychiatric patients experience more psychiatric symptoms during COVID-19 pandemic and lockdown? A case-control study with service and research implications for immunopsychiatry. Brain Behav Immun. (2020) 87:100–6. 10.1016/j.bbi.2020.04.069 32353518 PMC7184991

[19] Iob E Steptoe A Fancourt D. Abuse, self-harm and suicidal ideation in the UK during the COVID-19 pandemic. Br J Psychiatry. (2020) 217:543–6. 10.1192/bjp.2020.130 32654678 PMC7360935

[20] Torous J Jan Myrick K Rauseo-Ricupero N Firth J. Digital mental health and COVID-19: using technology today to accelerate the curve on access and quality tomorrow. JMIR Ment Health. (2020) 7:e18848. 10.2196/18848 32213476 PMC7101061

[21] Witt A Ordóñez A Martin A Vitiello B Fegert JM. Child and adolescent mental health service provision and research during the Covid-19 pandemic: challenges, opportunities, and a call for submissions. Child Adolesc Psychiatry Ment Health. (2020) 14:19. 10.1186/s13034-020-00324-8 32411296 PMC7213544

[22] Nittari G Khuman R Baldoni S Pallotta G Battineni G Sirignano A. Telemedicine practice: review of the current ethical and legal challenges. Telemed J E Health. (2020) 26:1427–37. 10.1089/tmj.2019.0158 32049608 PMC7757597

[23] Sampaio M Haro MVN De Sousa B Melo WV Hoffman HG. Therapists make the switch to telepsychology to safely continue treating their patients during the COVID-19 pandemic. Virtual reality telepsychology may be next. Front Virtual Real. (2021) 1:576421. 10.3389/frvir.2020.576421 33585834 PMC7880047

[24] Little H Tickle A das Nair R. Process and impact of dialectical behaviour therapy: a systematic review of perceptions of clients with a diagnosis of borderline personality disorder. Psychol Psychother. (2018) 91:278–301. 10.1111/papt.12156 29034599

[25] Neacsiu AD Rizvi SL Linehan MM. Dialectical behavior therapy skills use as a mediator and outcome of treatment for borderline personality disorder. Behav Res Ther. (2010) 48:832–9. 10.1016/j.brat.2010.05.017 20579633 PMC2914145

[26] Zeifman RJ Boritz T Barnhart R Labrish C McMain S. The independent roles of mindfulness and distress tolerance in treatment outcomes in dialectical behavior therapy skills training. Personal Disord. (2019) 11:181–90. 10.1037/per0000368 31647267

[27] Linehan MM Korslund KE Harned MS Gallop RJ Lungu A Neacsiu AD. Dialectical behavior therapy for high suicide risk in individuals with borderline personality disorder: a randomized clinical trial and component analysis. JAMA Psychiatry. (2015) 72:475–82. 10.1001/jamapsychiatry.2014.3039 25806661

[28] Landes SJ Chalker SA Comtois KA. Predicting dropout in outpatient dialectical behavior therapy with patients with borderline personality disorder receiving psychiatric disability. Borderline Personal Disord Emot Dysregul. (2016) 3:9. 10.1186/s40479-016-0043-3 27588205 PMC5007727

[29] Dixon LJ Linardon J. A systematic review and meta-analysis of dropout rates from dialectical behaviour therapy in randomized controlled trials. Cogn Behav Ther. (2020) 49:181–96. 10.1080/16506073.2019.1620324 31204902

[30] Bohnsack R. Rekonstruktive Sozialforschung: Einführung in qualitative Methoden. 5 ed. Opladen: Leske Budrich; (2003). 288 p. 10.1007/978-3-322-89614-8

[31] Mayring P. Qualitative Inhaltsanalyse. Grundlagen und Techniken. 12 ed. Weinheim: Beltz;. (2015) 152 p.

[32] Druss BG. Addressing the COVID-19 pandemic in populations with serious mental illness. JAMA Psychiatry. (2020) 77:891–2. 10.1001/jamapsychiatry.2020.0894 32242888

[33] Yao H Chen J-H Xu Y-F. Patients with mental health disorders in the COVID-19 epidemic. Lancet Psychiatry. (2020) 7:e21. 10.1016/S2215-0366(20)30090-0 32199510 PMC7269717

[34] Lakeman R Crighton J. The impact of social distancing on people with borderline personality disorder: the views of dialectical behavioural therapists. Issues Ment Health Nurs. (2020) 42:410–6. 10.1080/01612840.2020.1817208 32931341

[35] Vigerland S Lenhard F Bonnert M Lalouni M Hedman E Ahlen J. Internet-delivered cognitive behavior therapy for children and adolescents: a systematic review and meta-analysis. Clin Psychol Rev. (2016) 50:1–10. 10.1016/j.cpr.2016.09.005 27668988

[36] American Academy of Child and Adolescent Psychiatry (AACAP) Committee on Telepsychiatry and AACAP Committee on Quality Issues. Clinical update: telepsychiatry with children and adolescents. J Am Acad Child Adolesc Psychiatry. (2017) 56:875–93. 10.1016/j.jaac.2017.07.008 28942810

